# Evaluation of Allele Frequency Estimation Using Pooled Sequencing Data Simulation

**DOI:** 10.1155/2013/895496

**Published:** 2013-02-07

**Authors:** Yan Guo, David C. Samuels, Jiang Li, Travis Clark, Chung-I Li, Yu Shyr

**Affiliations:** ^1^Vanderbilt Ingram Cancer Center, Center for Quantitative Sciences, Nashville, TN, USA; ^2^Center for Human Genetics Research, Vanderbilt University Medical Center, Nashville, TN, USA; ^3^VANTAGE, Vanderbilt University, Nashville, TN, USA

## Abstract

Next-generation sequencing (NGS) technology has provided researchers with opportunities to study the genome in unprecedented detail. In particular, NGS is applied to disease association studies. Unlike genotyping chips, NGS is not limited to a fixed set of SNPs. Prices for NGS are now comparable to the SNP chip, although for large studies the cost can be substantial. Pooling techniques are often used to reduce the overall cost of large-scale studies. In this study, we designed a rigorous simulation model to test the practicability of estimating allele frequency from pooled sequencing data. We took crucial factors into consideration, including pool size, overall depth, average depth per sample, pooling variation, and sampling variation. We used real data to demonstrate and measure reference allele preference in DNAseq data and implemented this bias in our simulation model. We found that pooled sequencing data can introduce high levels of relative error rate (defined as error rate divided by targeted allele frequency) and that the error rate is more severe for low minor allele frequency SNPs than for high minor allele frequency SNPs. In order to overcome the error introduced by pooling, we recommend a large pool size and high average depth per sample.

## 1. Introduction

Over the last decade, large-scale genome-wide association studies (GWAS) based on genotyping arrays have helped researchers to identify hundreds of loci harboring common variants that are associated with complex traits. However, multiple disadvantages have limited genotyping arrays' ability for disease association detection. A major disadvantage of genotyping arrays is the limited power for detecting rare disease variance. Rare variants with minor allele frequency (MAF) less than 1% are not sufficiently captured by GWAS [1]. Such low MAF variants may have substantial effect sizes without showing Mendelian segregation. The lack of a functional link between the majority of the putative risk variants and the disease phenotypes is another major drawback for genotyping array-based GWAS [2]. The most popular genotyping chip, the Affymetrix 6.0 array, contains nearly 1 million SNPs, yet only one-third of these SNPs resides in the coding regions. Even though many GWAS-identified statistically significant SNPs lie in the intron or intergenic regions, [3–5] their biological function remains difficult to explain. Another limitation of genotyping arrays is that, because the SNPs are predetermined on the array, no finding of novel SNPs is possible.

Most of the above limitations can be overcome by using high throughput NGS technology [6]. NGS can target a specific region of interest, such as the exome or the mitochondria. Often, the functions of variants identified in coding regions of interest are much easier to explain than those of variants identified in the intron or intergenic regions. Also, by targeting the exome, we can effectively examine nearly 30 million base pairs in the coding region rather than just 0.3 million SNPs on the Affymetrix 6.0 array. Sequencing technology has been used to detect rare variants in many studies [7–10], with rare variants defined as 1%–5% frequency. Due to the large sample size needed to detect such low frequency variants, detection of rare variants less than 1% can still pose a significant challenge for NGS technology. One way to overcome this limitation is by doing a massive genotyping catalogue such as the 1000 Genomes Project [11]. Researchers are often too limited financially to conduct a genotyping study on such a large scale. DNA pooling is a strategy often used to reduce the financial burden in such cases.

The concept of pooling in genetic studies began in 1985 with the first genetic study to apply a pooling strategy [12]. Since then, pooling has been extensively applied in linkage studies in plants [13], allele frequency measurements of microsatellite markers and single nucleotide polymorphisms (SNPs) [10, 14–18], homozygosity mapping of recessive diseases in inbred populations [19–22], and mutation detection [23]. Even though pooling has also been used widely with NGS technology [24–26], the effectiveness of the pooling strategy has long been debated. On the one hand, several studies have claimed that data generated from pooling studies are accurate and reliable. For example, Huang et al. claimed that the minor allele odds ratio estimated from pooled DNA agreed fairly well with the minor allele odds ratio estimated from individual genotyping [27]. Docherty et al. demonstrated that pooling can be effectively applied to the genome-wide Affymetrix GeneChip Mapping 500 K Array [28]. Some studies have even found that pooling designs have an advantage in the detection of rare alleles and mutations, such as the study by Amos et al., which suggested that mutations in individuals could be more efficiently detected using pools [23]. On the other hand, several studies have argued that, when compared with individual sequencing, pooled sequencing can generate variant calls with high false-positive rates [29]. Other studies also found that the ability to accurately estimate the allele frequency from pooled sequencing is limited [30, 31].

Usually two different kinds of pooling paradigms are involved. The first is multiplexing (also known as barcoding). On an Illumina HiSeq 2000 sequencer, one lane can generate, on average, from 100 to 150 million reads per run. For exome sequencing, from 30 to 40 million reads per sample are needed to generate reliable coverage in the exome for variant detection. Thus, the common practice is to multiplex from 3 to 4 samples per lane to reduce cost. Using multiplexing with barcode technology, we are able to identify each read's origination. The disadvantage of multiplexing with barcoding is the extra cost of barcoding and labor. The cheaper alternative to pooling with multiplexing is pooling without multiplexing, which prevents us from identifying the origin of each read.

In this study, we focused on pooling without multiplexing. By using comprehensive and thorough simulations, we tried to determine the effectiveness of estimating allele frequency from pooled sequencing data. In our simulation model we considered important factors of pooled sequencing, including overall depth, the average depth per sample, pooling variation, sampling variation, and targeted minor allele frequency (MAF). Another important issue we addressed in our simulation is the reference allele preferential bias, which is a phenomenon during alignment when there is preference toward the reference allele. We used real data to show the effect of reference allele bias and adjusted our simulation model accordingly. We describe our simulation model in detail and present the results from the simulation.

## 2. Materials and Methods

We designed a thorough simulation model to closely reflect the real-world pooled sequencing situation. Our simulation model includes notations which we have defined as follows: let $\hat{F}$ be the allele frequency estimated from pooled sequencing data, and let *F* be the true allele frequency in the pool. Under the ideal assumption, all samples' contributions to the pool are equal. However, in practice, factors such as human error and library preparation variation can affect a sample's contribution to the pool. Very likely, each time a sample is added to the pool, an error is introduced. We let *ε*_*i*_ denote the error of sample *i* during the pooling process, and *ε*_*i*_ should follow a normal distribution *N*(*µ*, *σ* ^2^), where *µ* = 0, and *σ* ^2^ denotes the variance of error in the pool. We assume that the amount of DNA added to the pool for each sample *c* + *ε*_*i*_ follows a normal distribution *N*(*c*, *σ* ^2^), where *c* is a constant and denotes the ideal constant contribution to the pool by each sample. The probability that a read is contributed by sample *i* can be represented as *p*_*i*_ = (*c* + *ε*_*i*_)/∑_*i*=1_^*N*_*s*_^(*c* + *ε*_*i*_), where *N*_*s*_ denotes the number of samples in the pool. The contribution of each sample in the pool to a SNP can be modeled as a multinomial distribution *R*_1_, *R*_2_, *R*_3_, …, *R*_*N*_*s*__ ~ *Multinomial*(*D*, *p*_1_, *p*_2_, *p*_3_, …, *p*_*N*_*s*__), where *D* equals the depth at this SNP and *R*_*i*_ represents the reads contributed by sample *i* for this SNP. The depth *D* follows a possion distribution *Poisson*(*λ*_*D*_), where *λ*_*D*_ equals the average depth for the exome regions. For sequencing data, the reads at heterozygous SNPs should have an allele balance of 50%, meaning 50% of the read should support the reference allele while the other 50% of the read should support the alternative allele. Thus the reads that support the alternative allele should follow a binomial distribution *Binomial*(*D*, 0.5).

In our study we estimated the average depth for the exome regions as follows:

(1) Average  depth   =Lane×Reads  per  lane×Capture  efficiencynumber  of  exons.

In general the read output for 1 lane on an Illumina HiSeq 2000 sequencer is around 120 million reads. The most popular exome capture kits including Illumina TruSeq, Agilent SureSelect, and NimbleGen SeqCap EZ capture almost 100 percent of all known exons (about 30 million base pairs). Most capture kits claim that they have capture efficiency of at least 70 percent, but, in practice, it has been shown that the capture efficiency of all these capture kits are only around 50 percent [32], which implies that if a sample is sequenced for 120 million reads, only around 60 million reads will be aligned to exome regions. After filtering for mapping quality, the number of reads aligned to exome regions will be even smaller. However, to simplify, we ignored the reads that failed the mapping quality filter. There are about 180,000 exons [33]. Based on (1), for exome sequencing on 1 Illumina HiSeq lane, the average depth is expected to be roughly 400.

To measure the accuracy of the allele frequency $\hat{F}$ estimated from pooled sequencing data, we computed the relative root mean square error (RMSE) as follows:

(2) $$\begin{array}{c} \frac{\sqrt[{2}]{\sum_{1}^{n}{\left({\hat{F}}_{i}-F\right)}^{2}/n}}{F}, \end{array}$$

where *n* is the number of simulations we performed to estimate the target allele frequency *F*. In our simulations, we set *n* = 10,000. Unlike the traditional RMSE, we divided it by the target allele frequency *F* to make the result relative to the allele frequency we were simulating, so we could compare RMSE for allele frequencies as small as 0.5% and as large as 50%.

Reference allele preferential bias is a phenomenon during alignment when there is preference toward the reference allele. Degner et al. described such bias in RNA-seq data [34]. To examine whether this bias also exists in DNAseq data, we measured allele balance (defined as reads that support the alternative allele divided by total reads) of three independent DNA sequencing datasets. The three datasets were sequenced at different facilities (Broad Institute, HudsonAlpha, Illumina), at different time points, and using different capture methods (Agilent SureSelect, Illumina TruSeq, and Array Based Capture). The theoretical allele balance for heterozygous SNPs should be around 50%. In real data, we observed that the mean allele balance for all heterozygous SNPs for all samples is 0.483 (range: 0.447–0.499) (Table 1). Thus, we modified our previously defined read distribution at heterozygous site *Binomial*(*D*, 0.5) to *Binomial*(*D*, *P*) where *P* follows a normal distribution *N*(*µ*_*P*_, *σ*_*p*_^2^), where *µ*_*P*_ and *σ*_*p*_^2^ are estimated by the empirical mean allele balance we observed in real data.

Three simulations were conducted to evaluate the accuracy of allele frequency estimation from pooled sequencing data. The detailed descriptions of the three simulations are as follows.

**Simulation**

The goal of Simulation 1 was to study the relationship between different levels of *ε* and relative RMSE under different pool sizes (*N*_*s*_ = 200, 400, 800, and 1600) and different MAF (MAF = 0.5%,  1%,  %5,  10%,  20%,  30%,  40%, and 50%). Each sample's DNA contribution *c* + *ε*_*i*_ to the pool follows a normal distribution *N*(*c*, *σ* ^2^). For simulation purpose, we set an arbitrary value *c* = 10 units; the actual value of *c* does not affect the outcome of the simulations, because the simulation merely scales around it. To best represent the scenario in practice, we used several different standard deviations values for the distribution of sample contribution to the pool. For the ideal situation, we set *σ* ^2^ to a very small number (10^−5^); then, we increase *σ* ^2^ to 1, 2, and 4 (10%, 20%, and 40% of *c*) to see the effect of larger error variance on the accuracy of allele frequency estimation using pooled sequencing data. Each allele frequency was simulated 10,000 times.

**Simulation**

The goal of Simulation 2 was to study the relationship between depth and relative RMSE. The average depth of exome coverage can be estimated using the number of lanes. Instead of looking directly at average depth in the exome regions, we looked at average depth per sample *λ*_*ps*_ = *λ*_*D*_/*N*_*s*_ (i.e., average depth divided by pool size). If the average depth of exome regions for the pool of 200 people is 600x, then the depth per sample is 3x. In this simulation, we used *λ*_*ps*_ = 0.5, 1, 2, 4, 6, 8, 10, 12, 14,16, 18, and  20, pool size *N*_*s*_ = 200, 400, 800, and 1600, and MAF = 0.5%, 1%, %5, 10%, 20%, 30%, 40%, and 50%. Each allele frequency was simulated 10,000 times.

**Simulation**

The goal of Simulation 3 was to determine the overall performance of a pooled exome sequencing study. In practice, we cannot measure a SNP 10,000 times and then compute the average allele frequency as we did in Simulations 1 and 2. We are limited with one measurement only at a given SNP. It is important that we look at the overall performance too rather than just at a single SNP. Based on the released data of the 1000 Genomes Project, we built an empirical MAF distribution. This distribution should represent an overall picture of MAF distribution in the population. A typical exome study will yield 10,000–100,000 SNPs after filtering, with the number of SNPs heavily dependent on the number of samples sequenced in the study. Following the empirical distribution of the MAF, we randomly drew 10,000 SNPs from this distribution to simulate an exome sequencing dataset and computed an overall error rate. The error rate is defined as $\left|\hat{F}-F\right|/((\hat{F}+F)/2)$. We further repeated this simulation 1000 times and computed the median error rates.

## 3. Results

**Simulation**

**Figure 1:**
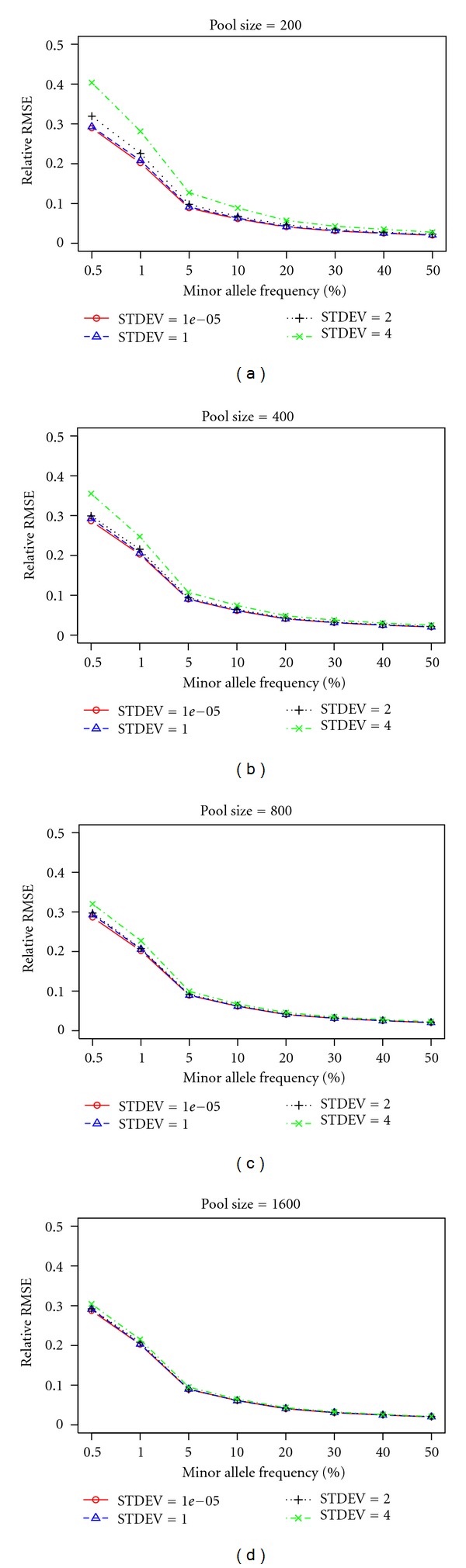
Relative RMSE for different pool sizes and MAFs under different standard deviations.

We assume that each sample's DNA contribution to the pool follows a normal distribution *N*(*c*, *σ* ^2^). In an ideal situation, *σ* ^2^ is small, and if we fix overall depth, the pool size does not make a significant difference for the RMSE. For example, in an ideal situation, for MAF = 0.5, the relative RMSE for pool size *N*_*s*_ = 200, 400, 800, and 1600 equals 0.023, 0.024, 0.024, and 0.020, respectively. However, if we increase *σ* ^2^, pools with greater size tend to have lower RMSEs. For example, when *σ* ^2^ is increased to 4, for MAF = 50%, the relative RMSEs for pool size *N*_*s*_ = 200, 400, 800, and 1600 equals 0.028, 0.0250, 0.023, and 0.022, respectively. Increasing *σ* ^2^ clearly also increased relative RMSE for all MAFs and for all pool sizes. For example, for pool size *N*_*s*_ = 200, MAF = 50%, *σ* ^2^ = 0.00001, 1, 2, and 4, the relative RMSE are 0.020, 0.021, 0.023, and 0.028, respectively. Also lower MAF tended to have high relative RMSE than high MAF. For example, in an ideal situation, for pool size *N*_*s*_ = 200, the relative RMSEs for MAF = 0.5%, 1%, 5%, 10%, 20%, 30%, 40%, and 50% are equal to 0.289, 0.202, 0.088, 0.061, 0.041, 0.031, 0.030, and 0.020, respectively. The results of Simulation 1 can be viewed in Figure 1.

**Simulation**

**Figure 2:**
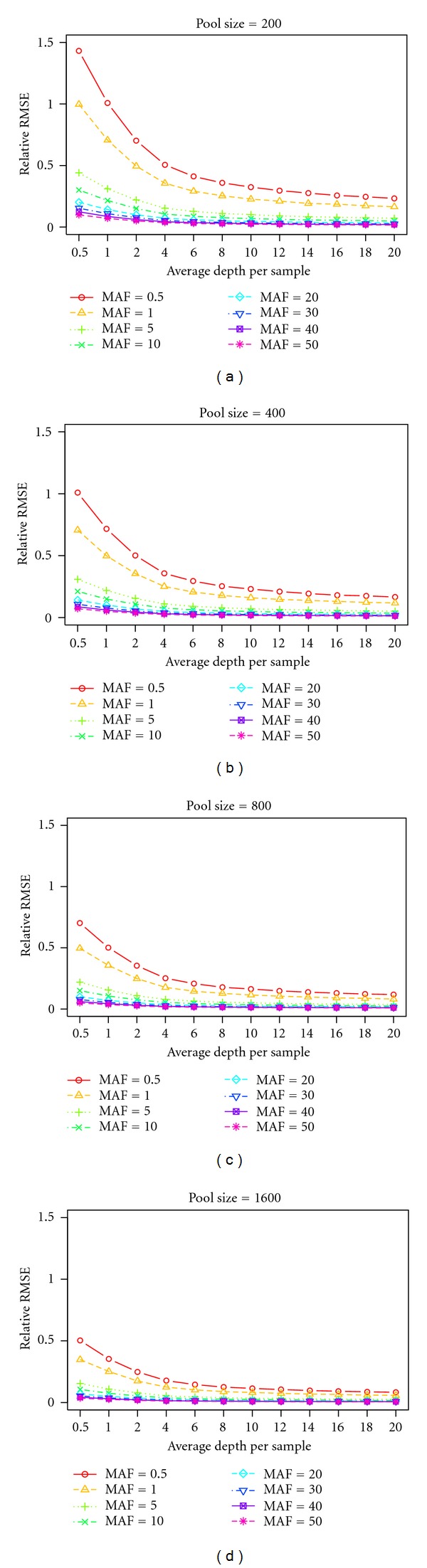
Relative RMSE for different pool sizes and MAFs under different average per sample depths.

In this simulation, the goal was to examine the relationship between average depth per sample *λ*_*ps*_ and pool size *N*_*s*_. We found that, with the same average depth per sample *λ*_*ps*_, higher pool sizes will generate lower relative RMSEs. Also, as the MAF increases, the relative RMSE decreases. For example, for MAF = 50% and average depth per sample *λ*_*ps*_ = 1, the relative RMSEs for *N*_*s*_ = 200, 400, 800, and 1600 are 0.071, 0.050, 0.036, and 0.025, respectively. If we can infinitely increase pool size or average depth per sample while fixing the other, the RMSE will reach zero. The result of Simulation 2 can be viewed in Figure 2.

In our study, we performed simulations at each MAF 10,000 times. However, in practice, we do not have the resources to measure a SNP 10,000 times and then take the average. In real exome sequencing, each SNP is only measured one time.  Table 2 shows the quantile information for simulating MAF = 0.5%, 1%, 5%, 10%, 20%, 30%, 40%, and 50% 10,000 times. The mean and median of the estimated MAF are very close to the targeted MAF value. When MAF increases, the variance also increases. If we account for relative RMSE, the simulations still produced more accurate results for larger MAFs.

**Simulation**

**Figure 3:**
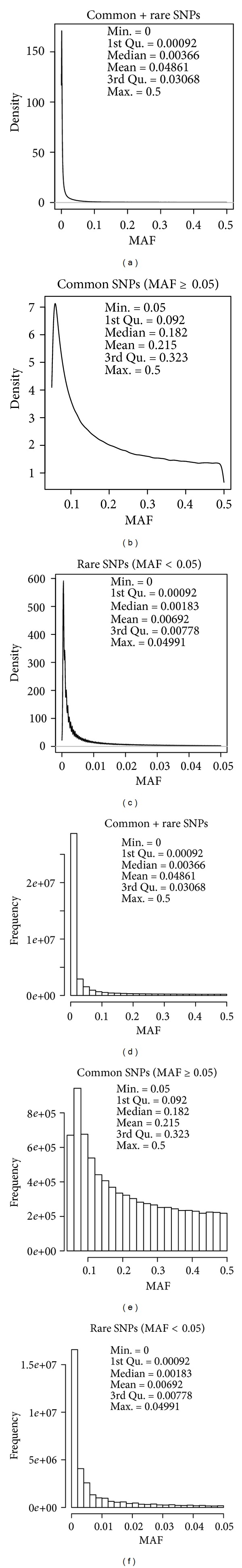
1000 Genome MAF distributions.

**Figure 4:**
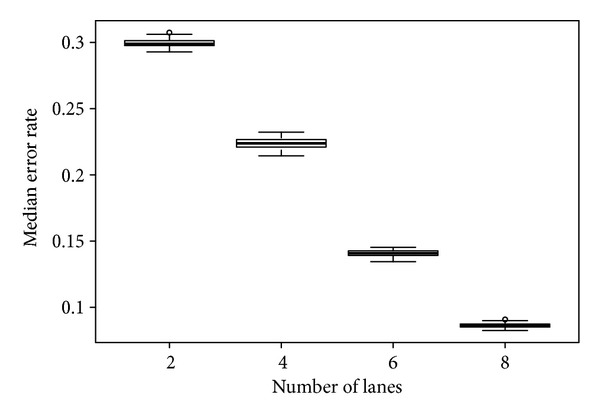
Median error rates for simulating 1000 exome sequences using different numbers of lanes. Simulation on 2 lanes shows nearly 30% error, and only around 5% error rate is observed for 16 lanes simulation.

In this simulation, we simulated the scenario of pooled exome sequencing. Using data from the 1000 Genomes Project as prior information that contains genotyping data from 1092 individuals, we built an empirical distribution of MAF (Figure 3). Based on this empirical distribution, we simulated the pooled exome sequencing with pool size *N*_*s*_ = 1092 1000 times and computed the median error rate for each simulation (Figure 4). The results clearly indicate that higher depth is required to produce an acceptable error rate (>5%). For standard exome sequencing, pooled DNA from 1000 subjects will require roughly 16 Illumina HiSeq lanes to produce results with an acceptable error rate.

*Financial Implication*. The ultimate goal of pooling is to ease the financial burden on large association studies. Based on the most up-to-date pricing information on NGS, we compared the total cost of conducting association studies using pooling at different pool sizes with individual sequencing using Illumina HighSeq 2000 sequencer, which contains 2 flow cells, and each flow cell contains 16 lanes.  Table 3 shows the price difference between pooling and individual sequencing. The savings using pooling is more substantial when pool sample size is large. When using all 16 lanes, the savings for 200 samples is roughly 500% over individual sequencing and, for 1000 samples, a 2300% saving.

## 4. Discussion

Our simulation showed that there are several important factors to consider when designing a pooling study. Those factors include sample size, targeted MAF, and, most importantly, the depth. The sample size directly affects the ability to detect rare SNPs. Larger pool size will increase the accuracy of MAF estimation with the same per sample depth but will not have much effect with the same overall depth. Similarly, with the same pool size, increasing depth will decrease relative RMSE. Our simulation also showed that pooled sequencing is not ideal for estimating the MAF of rare SNPs. The relative RMSE is much higher for SNPs with MAF < 1% compared to SNPs with MAF > 5% (Figure 1).

Sequencing pooled DNA will ease financial burdens and make large association possible. At the same time, however, pooling introduces additional errors. A majority of the errors are caused by the unequal representation of each sample's DNA in the pool. This unequal representation could be due to human or machine error, which we have considered in our simulation. There are other factors which can also cause the unequal representation, such as a sample's DNA quality and variation introduced in the PCR/amplification stage. Unfortunately, we can only minimize such errors and variation using more sophisticated lab techniques. Even if every sample is equally represented in the pool, the sequenced data still do not truly reflect the equality due to sampling variance. Based on our simulation results, when designing a pooling study, we recommend the following: larger pool size is better, and higher depth is better. More elaborately, it is better to keep balance between pool size and depth. We recommend keeping the average depth per sample at 10 minimum if rare SNPs are not of interest; otherwise, average depth per sample at 20 minimum is highly recommended.

## Figures and Tables

**Table 1:** Allele balance for 3 independent datasets.

Dataset	Sample	Min.	1st Qu.	Median	Mean	3rd Qu.	Max.	Mean 95% conf. lo.	Mean 95% conf. hi.
	1055QC0003	0.091	0.423	0.48	0.48	0.536	0.862	0.476	0.483
	1055QC0004	0.1	0.427	0.477	0.48	0.53	0.826	0.477	0.483
	1055QC0005	0.046	0.429	0.481	0.482	0.536	0.939	0.479	0.486
	1055QC0006	0.1	0.418	0.478	0.481	0.542	0.909	0.477	0.485
	1055QC0007	0.156	0.417	0.476	0.475	0.536	0.879	0.472	0.479
	1055QC0008	0.148	0.421	0.481	0.482	0.542	0.905	0.479	0.486
	1055QC0009	0.148	0.422	0.478	0.48	0.536	0.963	0.476	0.483
	1055QC0011	0.1	0.421	0.481	0.48	0.538	0.952	0.477	0.484
	1055QC0012	0.095	0.429	0.478	0.48	0.531	1	0.477	0.483
	1055QC0013	0.165	0.424	0.482	0.482	0.541	0.9	0.479	0.486
SureSelect	1055QC0014	0.103	0.429	0.481	0.483	0.538	0.818	0.48	0.487
	1055QC0016	0.13	0.425	0.48	0.482	0.54	0.909	0.478	0.485
	1055QC0017	0.136	0.422	0.481	0.48	0.536	0.9	0.477	0.483
	1055QC0018	0.182	0.424	0.48	0.48	0.537	0.987	0.477	0.483
	1055QC0020	0.2	0.432	0.483	0.485	0.536	0.815	0.482	0.488
	1055QC0021	0.12	0.429	0.481	0.484	0.538	1	0.48	0.487
	1055QC0022	0.091	0.424	0.478	0.479	0.533	0.905	0.476	0.482
	1055QC0024	0.077	0.422	0.478	0.478	0.535	0.857	0.474	0.481
	1055QC0025	0.13	0.429	0.481	0.484	0.54	0.897	0.481	0.488
	1055QC0026	0.13	0.42	0.478	0.479	0.539	0.793	0.476	0.482
	1055QC0028	0.039	0.419	0.477	0.476	0.531	0.938	0.472	0.479

	10009	0.044	0.447	0.5	0.499	0.55	1	0.496	0.501
	10244	0.091	0.444	0.5	0.497	0.55	0.909	0.495	0.499
TruSeq	10290	0.065	0.444	0.5	0.497	0.55	0.917	0.495	0.499
	20007	0.077	0.447	0.5	0.498	0.55	0.923	0.496	0.5
	20017	0.044	0.447	0.5	0.498	0.55	0.921	0.496	0.5
	20301	0.077	0.449	0.5	0.499	0.55	0.967	0.497	0.501

	ERR004043	0.04	0.376	0.44	0.447	0.511	0.986	0.44	0.453
	ERR004047	0.125	0.391	0.447	0.451	0.503	1	0.446	0.457
Array based	SRR013908	0.081	0.37	0.475	0.481	0.584	0.977	0.472	0.489
	SRR013909	0.071	0.372	0.476	0.484	0.591	0.95	0.476	0.492
	SRR015428	0.093	0.389	0.488	0.49	0.586	0.909	0.483	0.498
	SRR015429	0.1	0.426	0.496	0.497	0.564	0.913	0.491	0.503

All	Mean	0.103	0.421	0.482	0.483	0.543	0.919	0.479	0.487

**Table 2:** Statistics for doing 10,000 simulations at different MAFs.

MAF	Min.	1st Qu.	Median	Mean	3rd Qu.	Max.	Var.	Relative RMSE
0.5	0.0000	0.0036	0.0049	0.0050	0.0064	0.0162	0.0000	0.5037
1	0.0000	0.0075	0.0098	0.0100	0.0124	0.0264	0.0000	0.3552
5	0.0256	0.0448	0.0500	0.0500	0.0551	0.0795	0.0001	0.1540
10	0.0615	0.0928	0.0999	0.1000	0.1071	0.1401	0.0001	0.1070
20	0.1444	0.1904	0.2000	0.2001	0.2098	0.2558	0.0002	0.0716
30	0.2449	0.2889	0.2997	0.2998	0.3106	0.3619	0.0003	0.0537
40	0.3397	0.3879	0.3998	0.4000	0.4118	0.4707	0.0003	0.0442
50	0.4348	0.4877	0.5000	0.4998	0.5116	0.5675	0.0003	0.0359

**Table 3:** Pooled and individual sequencing pricing.

Sequencing per pool	200	400	600	800	1000
2 lanes	$3,650	$4,050	$4,450	$4,850	$5,250
4 lanes	$6,650	$7,050	$7,450	$7,850	$8,250
6 lanes	$9,650	$10,050	$10,450	$10,850	$11,250
8 lanes	$12,650	$13,050	$13,450	$13,850	$14,250
10 lanes	$15,650	$16,050	$16,450	$16,850	$17,250
12 lanes	$18,650	$19,050	$19,450	$19,850	$20,250
16 lanes	$24,650	$25,050	$25,450	$25,850	$26,250
Individual prep.	$125,000	$250,000	$375,000	$500,000	$625,000
